# Good practice in health care for migrants: views and experiences of care professionals in 16 European countries

**DOI:** 10.1186/1471-2458-11-187

**Published:** 2011-03-25

**Authors:** Stefan Priebe, Sima Sandhu, Sónia Dias, Andrea Gaddini, Tim Greacen, Elisabeth Ioannidis, Ulrike Kluge, Allan Krasnik, Majda Lamkaddem, Vincent Lorant, Rosa Puigpinósi Riera, Attila Sarvary, Joaquim JF Soares, Mindaugas Stankunas, Christa Straßmayr, Kristian Wahlbeck, Marta Welbel, Marija Bogic

**Affiliations:** 1 Unit for Social and Community Psychiatry, London and the Barts School of Medicine and Dentistry, Queen Mary University of London, Newham Centre for Mental Health, London, E13 8SP, UK; 2 Institute of Hygiene and Tropical Medicine, Universidade Nova de Lisboa, Rua da Junqueira, 96, 1349-008 Lisbon, Portugal; 3 Public Health Agency for the Lazio Region, Via S. Costanza 53, 00185 Rome, Italy; 4 Etablissement public de santé Maison Blanche, 3-5 rue Lespagnol, 75020 Paris, France; 5 Department of Sociology, National School of Public Health, 196 Alexandras avenue, Athens 11521, Greece; 6 Clinic for Psychiatry and Psychotherapy, Charité - University Medicine Berlin, CCM, Charitéplatz 1, 10117 Berlin, Germany; 7 Danish Research Centre for Migration, Ethnicity and Health (MESU), Unit of Health Services Research, Department of Public Health, University of Copenhagen, Øster Farimagsgade 5, DK-1014 Copenhagen, Denmark; 8 International and Migrant Health, NIVEL (Netherlands Institute for Health Services Research), Otterstraat 118-124, PO Box 1568, 3500 BN Utrecht, The Netherlands; 9 Institute of Health and Society, Catholic University of Louvain, Clos Chapelle aux Champs 30.05., 1200 Brussels, Belgium; 10 Agency of Public Health of Barcelona, Pça. Lesseps, 1, 08023 Barcelona, Spain; 11 Faculty of Health Sciences at Nyíregyháza, University of Debrecen, Sóstói út 31/B, 4400 Nyíregyháza, Hungary; 12 Department of Public Health Sciences, Section of Social Medicine, Karolinska Institutet, SE- 171 76 Stockholm, Sweden; 13 Department of Health Management, Lithuanian University of Health Sciences, A. Mickevičiaus g. 9, LT 44307, Kaunas, Lithuania; 14 Ludwig Boltzmann Institute for Social Psychiatry, Lazarettgasse 14A-912, 1090 Vienna, Austria; 15 National Institute for Health and Welfare (THL), Department for Mental Health and Substance Abuse Services, P.O.B. 30, FIN-00271 Helsinki, Finland; 16 Institute of Psychiatry and Neurology, Ul. Sobieskiego 9, 02-957 Warsaw, Poland

## Abstract

**Background:**

Health services across Europe provide health care for migrant patients every day. However, little systematic research has explored the views and experiences of health care professionals in different European countries. The aim of this study was to assess the difficulties professionals experience in their service when providing such care and what they consider constitutes good practice to overcome these problems or limit their negative impact on the quality of care.

**Methods:**

Structured interviews with open questions and case vignettes were conducted with health care professionals working in areas with high proportion of migrant populations in 16 countries. In each country, professionals in nine primary care practices, three accident and emergency hospital departments, and three community mental health services (total sample = 240) were interviewed about their views and experiences in providing care for migrant patients, i.e. from first generation immigrant populations. Answers were analysed using thematic content analysis.

**Results:**

Eight types of problems and seven components of good practice were identified representing all statements in the interviews. The eight problems were: language barriers, difficulties in arranging care for migrants without health care coverage, social deprivation and traumatic experiences, lack of familiarity with the health care system, cultural differences, different understandings of illness and treatment, negative attitudes among staff and patients, and lack of access to medical history. The components of good practice to overcome these problems or limit their impact were: organisational flexibility with sufficient time and resources, good interpreting services, working with families and social services, cultural awareness of staff, educational programmes and information material for migrants, positive and stable relationships with staff, and clear guidelines on the care entitlements of different migrant groups. Problems and good care components were similar across the three types of services.

**Conclusions:**

Health care professionals in different services experience similar difficulties when providing care to migrants. They also have relatively consistent views on what constitutes good practice. The degree to which these components already are part of routine practice varies. Implementing good practice requires sufficient resources and organisational flexibility, positive attitudes, training for staff and the provision of information.

## Background

Migration to European countries has increased since 2000 [1]. Estimates suggest that in 2005, 8.5% of the EU population consisted of migrants, with an additional 5.6 million arriving between 2005 and 2009 [2]. A central challenge for Europe, with its increased proportion of migrants, is the provision of accessible, equitable, and good quality health services for all.

Most research on health care for migrants has focused on access and use of services by migrants, with much of the literature coming from the US [3,4], Canada [5], Spain, [6-8], Denmark [9], the UK [10] and Sweden [11,12]. Studies suggest that migrants experience unequal access to care [13], and outline the complexity of health care entitlements of migrants [14-16].

This study did not address the problems of accessing care, but the quality of care provided to migrants once they are in a service, an issue which has received comparatively less attention in the literature, but is of central importance to millions of migrants who are treated in health services across Europe every day. Numerous health care professionals in different European countries face the challenge of providing the best possible care to migrant patients, and many of them have a wealth of experience [17,18]. Yet, there has been little systematic research exploring their views and experiences on what they see as the problems and what they regard as good practice.

This study explored the views and experiences of those health care professionals in different types of services across Europe, who provide care to migrants on a daily basis. The aim was to assess what problems they experience in their service, and what they view as good practice to overcome these problems or limit their negative impact on the quality of care.

## Methods

### Recruitment and Sampling

As part of the EC funded project 'Best Practice in Health Care Services for Immigrants in Europe' (EUGATE) [19] interviews were conducted with health care professionals in 16 European countries (covering more than 85% of the EU population) to identify their experiences and views of providing health care to migrants.

To ensure the participants had sufficient experience of providing health care to migrants, each participating country was asked to identify and recruit participants from areas where migrant population levels were particularly high. The plan was to focus on the three districts in each capital, or another urban context, with the highest proportion of immigrants.

A refined definition of migrants was used to aid classification of areas high in migrant groups. Migrants were defined as persons who were born outside the country of current residence, and who were aged between 18 and 65 years. In line with EU directives; regular immigrants (e.g. labour immigrants), refugees, asylum seekers, victims of human trafficking, and illegal/undocumented immigrants were all encapsulated in this definition.

Once areas were identified, for each of the three districts in each country, participants were recruited from three primary care practices (i.e. 9 per country, total n = 144), and one each from an accident and emergency hospital department (3 per country, total n = 48), and a community service for patients with mental illnesses (3 per country, total n = 48). In selecting primary care practices we aimed to include those with the highest number of migrant patients or, in the absence of such data, the biggest ones in the area. Whilst usually there was only one A&E department in each area, for community mental health services we aimed to select the largest one in the given area. The list of selected areas and health service in all 16 countries are shown in Table 1.

**Table 1:** Sampled countries and corresponding health service areas

Country	Selected Cities	Selected Primary Care Areas	Selected Mental Health Care Areas	Selected A & E Areas
AUSTRIA	Vienna	Rudolsheim-Fünfhaus, Ottakring, & Brigittenau.	Rudolsheim-Fünfhaus, Ottakring, & Brigittenau.	Leopoldstadt, Ottakring, & Brigittenau.

BELGIUM	Brussels	Saint Josse, Schaerbeek, & Molenbeek	Saint Josse, Schaerbeek, & Molenbeek	Saint Josse, Schaerbeek, & Brussels City

DENMARK	Copenhagen	Nørrebro, Valby, Bispebjerg, Brøndby, Albertslund, & Rødovre	Areas within Bispebjerg Hospital, Hvidovre Hospital, & Glostrup Hospital	Bispebjerg Hospital, Hvidovre Hospital, & Glostrup Hospital

FINLAND	Vaasa, Pietarsaari, Oravais, Malax	Vaasa, Pietarsaari, & Oravais,	Vaasa, Pietarsaari, & Malax	Vaasa, Pietarsaari, & Oravais,

FRANCE	Paris	18^th ^& 19^th ^Arrondissments of Paris & Aubervilliers of Seine-St-Denis Department	18^th ^& 19^th ^Arrondissments of Paris & Aubervilliers of Seine-St-Denis Department	Bichat, Lariboisiere, & La Roseraie

GERMANY	Berlin	Tiergarten, Wedding, & Kreuzberg	Tiergarten, Wedding, & Kreuzberg	Tiergarten, Wedding, & Kreuzberg

GREECE	Athens	Vari, Vyronas, Galatsi, Thrakomakedones, & Elefsina	Central Athens	Voula & Thrakomakedones

HUNGARY	Budapest	Terézváros, Erzsébetváros, Kőbánya, Zugló, & Csepel	Erzsébetváros, Kőbánya, & Pesterzsébet	Erzsébetváros, Kőbánya, & Pesterzsébet

ITALY	Rome	Districts I, VIII, & XX	Districts I, VIII, & XX	Districts I, VIII, & XX

LITHUANIA	Kaunas	Nine largest PHC out of 11	Aleksotas & Zaliakalnis	Downtown Kaunas, Zaliakalnis, & Silainiai district

NETHERLANDS	Amsterdam, Utrecht, Rotterdam, Hague	Amsterdam, Utrecht, & Rotterdam	Amsterdam, Utrecht, & Rotterdam	Amsterdam, Utrecht, & the Hague

POLAND	Warsaw	Mokotow, Praga Poludnie, & Srodmiescie	Mokotow, Praga Poludnie, & Srodmiescie	Mokotow, Praga Poludnie, & Srodmiescie

PORTUGAL	Lisbon	Amadora, Loures, & Lisboa	Amadora, Loures, & Lisboa	Amadora, Loures, & Lisboa

SPAIN	Barcelona	Cuitat Vella, Eixample, Sants Montjuic, & Nou Barris	Cuitat Vella, Eixample, & Nou Barris	Cuitat Vella, Eixample,& Nou Barris

SWEDEN	Stockholm	Central, South East, & South West	Central, South East, & South West	Central, South East, & South West

UNITED KINGDOM	London	Hackney, Tower Hamlets & Newham	Hackney, Tower Hamlets & Newham	Hackney, Tower Hamlets &Newham

We approached the selected service and asked for an interview, preferably with a practitioner with the largest experience in providing health care to migrants in the service. The decision as to who was to be interviewed in the study was made by the service.

### The Interviews

Face-to-face interviews were conducted between 2008 and 2010. A structured interview schedule was developed in English and piloted in each participating country. Based on the experiences of the pilot phase, the schedule was refined and finalised. The final version of the schedule was translated into the languages of the participating countries. It was presented in two parts. The first part of the interview focused on open questions about general experiences, in particular problems and strengths in providing health care to migrants within the service. The second part consisted of open-ended questions about patients represented in three case vignettes. These cases were modified to suit the three types of services (primary care, A&E, and community mental health) and aimed to specify differences and similarities in treatment for migrants in comparison to indigenous populations (as can be seen in the text from the vignettes below).

#### Primary Care

##### Illegal immigrant

A male, 28 years old, coming from [insert country], presents with pain when urinating and has a slight fever. He does not speak any language that the doctor understands. He has no insurance, no identification and no residency permit.

##### Refugee

A refugee woman, 39 years old, from [insert country], presents with headache, anxiety, sleeping problems and stomach ache. She has very little command of the language of the host country. She brings her 12 years old daughter along, who speaks the language of the host country very well.

##### Labour immigrant

A [insert nationality] woman, 40 years old, labour immigrant, widow, with two children 10 and 15 years old, asks for medication for her lower back pain. She speaks the language of the host country reasonably well, is working legally in a cleaning company and wants to go back to work urgently.

#### Accident & Emergency Department

##### Illegal immigrant

The patient arrived in the host country as an illegal immigrant about 1 year ago. He is 25 yrs of age and of *[insert a country] *origin. He does not speak any language that the A&E staff understands and presents with an intense lower abdominal pain.

##### Refugee

The female patient is 19 yrs of age and arrived from *[insert a country] *10 months ago. She has refugee status and speaks only *[insert language of origin] *and a few words of English. She is in her fifth month of pregnancy and has a serious complication (pre-eclampsia). She is reluctant to be examined by a male doctor.

##### Labour immigrant

The male patient is 35 yrs of age and arrived from *[insert a country] *two years ago. He has a regular residence permit. He was brought to A&E by the police because of his aggressive behaviour following heavy drinking. He suffered external head injuries in a fight. He is fully conscious and accessible for examination.

#### Mental Health Services

##### Illegal immigrant

The patient arrived in the host country as an illegal immigrant about 1 year ago. She is 25 yrs of age and of *[insert nationality] *origin. She does not speak the language of the host country, has no social contacts and appears severely depressed.

##### Refugee

The male patient is 22 years of age, came to the host country from *[insert country] *a year ago and has refugee status. He speaks a few words of the language of the host country. He appears to have persistent auditory hallucinations and feels persecuted.

##### Labour immigrant

The female patient is 45 yrs of age and arrived from *[insert country] *two years ago. She has a regular residence permit, speaks the language of the host country well and suffers from a bipolar disorder with frequent and prolonged manic episodes.

The country of origin of the migrants in the vignettes varied slightly between countries to ensure that the origin of the migrant was consistent with the demographic composition of migrants in the participating country, so that the vignettes presented a realistic scenario to the interviewees in each country. In all other ways, the case vignettes and questions were identical across countries.

Demographic information and professional standing of the interviewees were documented and the interviews were audio-taped in the majority of cases. When this was not possible, responses were documented in writing. Informed consent was obtained prior to the interviews, and the study was approved by relevant ethics committees in countries where this was required. Ethics approval for the study was obtained in Portugal through the University Hospital S. João. In other countries ethics approval was not required because no patient data was recorded, because the study was regarded as service evaluation without the need for an ethical review.

### Data Analysis

All of the 240 interviews were audio-taped or recorded in writing and transcribed verbatim, ensuring the removal of any identifying information to maintain anonymity. The prepared transcripts were subjected to thematic content analysis [20]. This process was deemed the most suitable for interpreting the textual data in a systematic way, from classifying codes to identifying emergent themes.

The first stage was to code the data from the initial interview transcripts line-by-line, which was conducted in each of the participating centres. The codes and corresponding textual extracts were then used to develop a codebook, which was translated into English, reviewed and finalised amongst the researchers in all participating countries. The resulting codebook consisted of a list of codes, accompanied by a brief and a more comprehensive definition, and illustrated with examples. The codebook was utilised to code the entire data corpus, with strategic checks made after coding of the first six interview transcripts from each country. The validity of the codes was checked against the data extracts to ascertain grounding in the transcribed data. Discrepancies in coding across centres were picked up early in the analysis and clarified with verifications, further to those summarised in the codebook. The second stage of the analysis was the clustering of codes into emergent categories, which were then structured and grouped to form overarching themes [21,22]. To ensure the themes and categories consistently represented the data corpus across all participating centres, final verification checks were made between each participating centre and the coordinating centre.

Data from all parts of the interview and from all countries were included in frequency counts of themes. This numerical depiction of content was used to specify the frequency of the problems and good practice components and compare those frequencies across services.

## Results

### Participants

The majority of interviewees were practitioners (n = 214) including doctors (156), nurses (44), psychologists (7), physiotherapists (4) and social workers (3). The remaining participants were either administrators or managers, some of whom were also qualified health care professionals (n = 26).

### Differences in further treatment for migrants

The majority of respondents (74%) asserted that, in general, treatment for migrants after the initial contact would not differ from that for non-migrant patients. When specifically asked in case vignettes about different further pathways depending on the immigration status, for the labour migrant vignette over two-thirds (147 participants) explicitly said that there would be no difference in further treatment pathways. However, for refugees and undocumented migrants only one or two participants respectively reported no difference in further treatment pathways.

### Problem Areas

Eight problem areas were identified, which are listed in Table 2. These are comprehensive and include all problems mentioned in the 240 interviews. The problems are here presented in the order of the frequency of interviews in which they have been raised.

**Table 2:** Frequency of problem areas reported amongst interviewees by service type

Themes	Service Type (%)			Totals (%)
**Problem Areas**	**Primary Care**	**Mental Health**	**A&E**	**All Services**

Language barrier	137 (95)	45 (94)	46 (96)	228 (95)

Difficulties in arranging care for migrants without health care coverage	124 (86)	33 (69)	28 (58)	185 (77)

Social deprivation and traumatic experiences	101 (70)	41 (85)	26 (54)	168 (70)

Lack of familiarity with the health care system	92 (64)	27 (56)	31 (65)	150 (63)

Different understandings of illness and treatment	79 (55)	36 (70)	24 (50)	139 (58)

Cultural differences	74 (51)	26 (54)	38 (79)	138 (58)

Negative attitudes among staff and patients	58 (40)	21 (44)	21 (44)	100 (42)

Lack of access to medical history	24 (17)	10 (21)	13 (27)	47 (20)

#### 1) Language barrier

Language and communication problems were most commonly reported, with frequent references made to a 'language barrier' between practitioners and patients. Concerns were expressed for migrants' inability to communicate their problems due to language difficulties, with the risk of being misunderstood and, ultimately, misdiagnosed. Respondents described how extensive physical examinations and diagnostic tests were sometimes required to compensate for the inability to communicate verbally. Administrative procedures were also prolonged and complicated through poor communication.

Some interviewees outlined associated problems with no or restricted access to interpreting services, which often resulted in the use of the patient's child, or another family member translating during consultations. This was especially problematic in sensitive cases.

*"There is often a significant language barrier. If everything has to be translated, you lose half the time. Often a child or grandchild is translating, but then you can't ask personal intimate things anymore. A ten year old girl can't translate the menstruation problems of her mother. That's really a problem." *(Netherlands, ID 212, Primary Care)

Family members may also choose to be selective in what they translate, summarising or even censoring the communication between the patient and the doctor.

*"When it is a family member who comes to translate, he translates what he wants, it's only interpretation..." *(Belgium, ID 27, Primary Care)

Involving a professional interpreter however may also come with problems. Concerns were expressed for how involving a third party would impact on the patient-practitioner relationship. Third party involvement also led some participants to be concerned over confidentiality issues, especially when the interpreter was from the patient's own community.

#### 2) Difficulties in arranging care for immigrants without health care coverage

Respondents discussed the difficulties in providing care for undocumented immigrants, who had no entitlements to mainstream health care services. Some professionals reported that the entitlements of different patient groups required clarification. Others mentioned that they had sufficient information to know what treatments they could offer, where the patient could seek further help, or how the treatment should be funded. Awareness of the legal situation may put practitioners into a dilemma.

*"Unfortunately, sometimes even legal immigrants are not covered by general health care insurance. This is a big problem for doctors, because in theory, uninsured patients should cover the costs of their treatment by themselves. But for most immigrants it is impossible... And doctors are in a situation with no good solution - from an ethic point of view they should provide treatment, from a legal point of view - they shouldn't." *(Poland, ID 234, Primary Care).

Most interviewees said that they would always provide emergency care if required. They described restricted access to laboratorial tests, scanning and other specialist pathways for migrants without coverage. Some interviewees attempted to circumnavigate the coverage problems by submitting laboratory samples in their own name, prescribing the patient with a cheaper medicine they could afford, or choosing to register the patient in an alternative manner. Some interviewees expressed concern that they would not be able to contact the patient again if tests raised abnormal results, or that migrants fearful of deportation would risk using fake identification or someone else's documents to receive care.

#### 3) Social deprivation and traumatic experiences

Over two-thirds of the interviewees reported problems arising from stressful experiences for migrants. Recent migrant patients were viewed as being more socially marginalised, from poorer backgrounds, unemployed, struggling to learn a new language, or to integrate, and possibly traumatised from experiences of war and conflict.

"*...that lady from the Congo had her foot sawn off as a form of torture. Other things like that, multiple rape, people who have had their lips cut off, or their whole family murdered in front of them..." *(UK, ID 305, Primary Care)

Some of these specific socioeconomic stressors had a direct impact on treatment.

*"...the difference is, that there is more [treatment] and less prevention. That I just can't put her on sick-leave, that I can't advise her to change her job - how should she attend a training, and let her children starve, that is not possible and that is the difference." *(Austria, ID 2, Primary Care).

Some respondents held the view that resolving socioeconomic and legality issues were of more importance to many patients than resolving health problems.

#### 4) Lack of familiarity with the health care system

A lack of familiarity with the health care system was regarded as common among recent immigrants.

"*A&E services are often the only care access many migrants have - because they don't know how the system works." *(France, ID 806, A&E).

Not fully understanding the health care system affects the treatment available. Interviewees reported cases where available resources and services were underused by migrants, because they were not aware of their existence. Furthermore, respondents discussed that previous experience in other health care systems often led migrants to have different expectations of the roles of doctors and patients. Different understandings of the patient-clinician relationship may result in uncertainty and mistrust, if experiences differ greatly from expectation. Interviewees regarded the role of doctors as given greater precedence amongst certain migrant patients, who may have unrealistic expectations about the capacity of doctors to sort various physical and social problems within short consultations.

#### 5) Different understandings of illness and treatment

Participants reported problems linked specifically to different understandings of the given illness of a migrant patient and the treatment options. Expressions of aetiology, symptoms, and pain made a diagnosis difficult to establish, especially when understandings of these concepts greatly differed between the patient and practitioner.

Respondents discussed the challenges in treating migrant patients with different understandings of the human body, which occasionally resulted in patients deciding not to follow the recommended treatment, or agreeing after some resistance.

*"I had this woman from Somalia who said her back was hurting and her understanding of the pain was that she had some air which was moving from one side of the back to the other *[...] *she wanted me to perforate the shoulder so that the air could get out. It was very difficult to explain why I just gave her tablets because her perception of her body is completely different. [...] Even with an interpreter it was very difficult to explain so we had to find my anatomy book and show [...] her problem with the back was with muscles and that there was no air here. She kind of understood though she did not look completely convinced, but she took the pills and it helped." *(Denmark, ID 49, Primary Care)

#### 6) Cultural differences

Whilst the previous problem was specifically linked to the understanding of the given illness and its treatment, interviewees reported also more general differences in cultural norms, religious practices and customs as potential complications to direct examination and treatment. Interviewees reported concerns regarding appropriate engagement in physical examinations, preserving and respecting religious restrictions on physical contact and cultural taboos.

*"...members of Muslim religious communities, there are shame barriers that we do not have: the husband expects to attend the treatment session. In certain treatment- the areas of sex, anal region are taboo." *(Germany, ID 101, A&E)

While most services were able to offer treatment from either gender if requested, others were not. According to the respondents, this had on occasion resulted in patients refusing care or unwilling to disclose sensitive information.

Interviewees noted that some European treatments and traditions may be difficult for migrants to embrace, particularly when they involve therapies and treatments outside of medication.

*"Different cultural values and beliefs make it difficult for the doctor to use psychotherapeutic procedures" *(Greece, ID 122, Mental Health).

Respondents also discussed cultural differences in terms of practical issues such as not attending appointments, turning-up late, or seeking consultation outside of opening hours. Often this was discussed as leading to disappointment and frustration, as patients would be asked to make another appointment. There were also concerns for the impact this would have on the service, with delays to other appointments, and a general strain on time and resources.

#### 7) Negative attitudes among staff and patients

Interviewees reported a lack of trust of some migrant patients towards staff. Distrust towards practitioners and interpreters originating from countries where patients previously experienced political or religious conflict were reported in this context. Certain patients were reported as being explicit in their requests to be seen by another member of staff, or withholding information, based on these grounds. Negative attitudes towards staff and sometimes hostile behaviour were largely attributed to cultural differences, misunderstandings, or the feeling of the patients that they were not being taken seriously.

Fears of discrimination were mentioned in explanations of patient reticence, often based on current and previous societal experiences, or opinions reported in the media. However, staff behaviour towards migrant patients may also perpetuate this fear of discrimination.

*"Many migrants experience discrimination and rejection within the healthcare system; being sent away, being treated unkindly, treated as if they are stupid, while they do not understand the language. These experiences are taken along in the doctor-patient relationship. I can notice the distrust of new clients at their first consultation with me." *(Netherlands, ID 214, Primary Care).

#### 8) Lack of access to medical history

Finally, lack of access to a medical history was reported as problematic, especially for undocumented migrants. If such information was available, it was usually in a foreign language. Respondents further discussed the complications associated with not knowing whether patients had allergies, vaccinations, or previous health problems. They were concerned that lack of contact details and nationality made decisions regarding consent and next of kin problematic.

#### Differences between types of services

Most problems were similarly raised in all three types of services. Primary care services more often mentioned difficulties in arranging further care, whilst community mental health services put more emphasis on the social stressors for migrants and A&E departments on different cultural norms. Negative attitudes and lack of access to medical history were raised as problems in 15 countries; the other problem areas were raised in all 16 countries.

### Components of good practice

Seven themes describing different components of good practice emerged and covered all components mentioned in the interviews. They are summarised in Table 3 and reported below in order of frequency. Statements on good practice were considered in the themes independently of whether respondents mentioned them as an existing strength of their service, or a suggestion for future improvements.

**Table 3:** Frequency of components of good practice reported amongst interviewees by service type

Themes	Service Type (%)			Totals (%)
**Components of Good Practice**	**Primary Care**	**Mental Health**	**A&E**	**All Services**

Organisational flexibility with sufficient time and resources	138 (96)	48 (100)	48 (100)	234 (98)

Good interpreting services	142 (99)	38 (79)	38 (79)	218 (91)

Working with families and social services	67 (47)	48 (100)	18 (38)	133 (55)

Cultural awareness of staff	58 (40)	32 (67)	22 (46)	112 (51)

Education programmes and information material for migrants	66 (46)	15 (31)	23 (48)	104 (43)

Positive and stable relationships with staff	62 (43)	13 (27)	16 (33)	91 (38)

Clear guidelines on care entitlements of different groups of migrants	15 (10)	8 (17)	4 (1)	27 (11)

#### 1) Organisational flexibility with sufficient time and resources

Almost all respondents mentioned aspects relating to organisational flexibility, including sufficient time, resources and individualisation of care.

Many practitioners reported booking double sessions, especially when an interpreter was involved, and giving migrant patients more time to ensure that they were heard and understood. Where limited time and resources were reported, the respondents suggested that staff could be employed to specifically manage social and administrative issues, freeing more time for practitioners to see patients in a health care capacity.

*"...to have more professionals with time available to provide information to these patients so they can feel that they have a place where they can go and ask their questions." *(Portugal, ID 244, A&E).

Interviewees discussed the importance of structuring regular staff meetings to deal with the problems arising in health care to migrants.

Some services faced restrictions on treating undocumented migrants. To overcome this barrier to further care, suggestions were made to seek funding for treatment from Non Governmental Organisations, sending patients to clinics specialising in providing care to undocumented migrants, providing cheap or free medication, giving private prescriptions, or registering undocumented migrants in an alternative way (e.g. as a tourist).

*"I prescribe the medicines for my own name, if the patient has no money for it." *(Hungary, ID 146, Primary Care).

In practice many respondents reported that staff would first treat patients and then possibly consider issues of entitlement and insurance. Respondents also mentioned that services to migrants could be improved with the use of more documentation, even for those with no legal residency. Recommendations were made for establishing databases with medical histories.

Several interviewees mentioned close geographical proximity to immigrant populations as a strength of their service. Providing a local service for migrants reduced problems associated with keeping appointments, and the cost of transportation.

Respondents spoke of the importance of a flexible and individualised approach for migrants within mainstream care, with more walk-in sessions, open appointment slots, and advocacy services.

#### 2) Good interpreting service

Good interpreting services were mentioned in almost all of the interviews and respondents were specific in what was required for a good quality interpreting service.

*"Qualitative interpreting services, so that the interpreter knows the medical terminology and also understands the professional discretion*" (Finland, ID 73, Primary Care).

They suggested that this could be achieved through professional interpreters, recommending improved access to interpreting services, including the availability of interpreters at the reception point and facilities for multiple languages. The provision for a permanent in-house interpreting service was discussed by some, as were same-language therapists for patients receiving talk-based therapies. Others emphasised improving migrant patients' command of the national language as the best possible long-term answer to reducing language barriers.

Communication through a professional interpreter was not always viewed as entirely helpful. Some interviewees preferred using relatives or friends as interpreters instead, because of their ability to provide more comprehensive information about the patient, as well as having the patient's trust. Respondents reported using the internet to assist in translation, by the use of search engines or web pages with medical advice and information from the patient's country of origin.

#### 3) Working with families and social services

Just over half of the respondents suggested collaboration with social services and families as important for good practice in migrant health care. Central to this theme were good contacts with social services and the sharing of information.

Interviewees explicitly mentioned engaging with community centres to connect migrant patients to the wider community. Some reported contacting religious leaders and non-statutory agencies to assist migrants in getting in touch with their local community. Concerns were also raised about migrants becoming isolated. Respondents addressed attempts made by health care staff in some services to contact the patient's family or friends, even if they were in another country.

Participants raised concerns that in some cases the patient's living conditions maybe exacerbating an illness or limiting recovery and discussed instances where they had attempted to find solutions to the patient's personal and social problems. For example, some referred patients with housing problems to charities with housing facilities. Other health services have Citizens Advice Bureau advisors, physiotherapists, cultural welfare advisors, and family action advisors to assist immigrants with different needs in one service. Respondents discussed the benefits of dealing with health, administrative and legal issues in one place. They reported often to encourage migrant patients to get in touch with refugee organisations, projects for immigrant women, language learning centres and other training courses.

#### 4) Cultural awareness of staff

Cultural awareness was reported as important for good practice. Some respondents viewed the training of staff in different cultural and religious practices as core to the delivery of satisfactory and respectful care to migrant patients. Some spoke of developing expertise in the treatment of migrant patients through experience and exposure, such as being located in a multicultural community, or being known as a culturally sensitive service.

Respondents made specific recommendations for topics on cultural sensitivity to be covered in practitioner training courses and university education. They further suggested that courses should include information on migrant specific diseases, cultural understandings of illness and treatment, and information pertaining to cultural and religious norms and taboos.

"*There are the lack of knowledge how to work with this type of patients. Doctors are lacking legal, cultural, specific medical information about this. It would be good to organize a short training course in this field." *(Lithuania, ID 186, A&E).

According to the respondents, such knowledge enabled them to reach more accurate diagnoses and provide appropriate treatments, while meeting patient needs for cultural acceptance and understanding. The presence of migrant staff was also flagged up as increasing the awareness of migrant needs and assisting with understanding culture and language issues.

"*It is a great strength that we have staff of different ethnic background. We can learn from them once in a while when there is an episode where we think "what just happened here?" Then it is a gift to have an employee who is able to say what they think it is about. And often it is. In acute situations we have a language which we need here and now..." *(Denmark, ID 44, Mental Health).

#### 5) Educational programmes and information material for migrants

Interviewees suggested that instructive programmes and information material be produced for migrants about the host country's health care system. Such information was viewed as helpful for migrants to access appropriate services and seeking effective treatment. Suggestions were made for community health projects, or evening meetings, where medical staff could explain and educate migrants about how the health care system works, and how to foster a healthy lifestyle.

*"They should design a welcome process adapted to immigrant people to explain the health care system, counselling etc. For example, they should have an interview with the immigrant patients to inform them about the health care system and the service roles." *(Spain, ID 265, Primary Care).

As one way of providing such information respondents suggested the use of leaflets in multiple languages, explaining the health care system and avenues for accessing services. Some interviews felt this took some pressure off practitioners, so that they would spend less time explaining the system and more time providing direct patient care. In addition, interviewees purported that this would reduce patient disappointment, as awareness of what can be expected from each service and staff would be unambiguous. However, some respondents cautioned that migrant patients may still need assistance to be guided to leaflets and, where literacy is an issue, more assistance would be required than just a leaflet.

#### 6) Positive and stable relationships with staff

Over a third of all respondents pointed towards positive relationships between staff and patients, and continuity of care as components of good practice. They discussed the necessary features for a positive relationship, which included respect, warmth, being welcoming, listening and responding effectively. Some respondents spoke of having welcoming policies in place, which ensured that patients are given individual attention and eased processes for them where possible. The promotion of non-judgmental, open minded and equitable staff was also mentioned in responses under this theme.

Consistency of staff was seen as important for achieving familiarity and building a positive and trusting relationship.

*"I have built a relationship with my clients, and have gradually come to know them. I am a familiar person, and they know they can always contact me. If I am absent, they can contact a colleague of mine, with whom they are also familiar." *(Netherland, ID 214, Primary Care).

Respondents further reported that seeing a different clinician at every appointment had a negative impact on patients' experience of the service, especially when they had to explain their medical history repeatedly in every consultation.

#### 7) Clear guidelines on care entitlements of different groups of migrants

Several respondents suggested clearer information and guidelines on what type of care different migrant groups are entitled to. They reported the benefits from courses on migrant health care rights and other legal issues. Included under this theme were suggestions for information on how to gain funding for treating undocumented migrants. Some governments legally allow practitioners to treat undocumented migrants if their condition was life threatening. However, respondents reported that transparency was needed on what was considered a life threatening condition.

#### Differences between types of services

Collaboration with families and social services was more often reported as important in community mental health services, whilst the other components of good practice were raised by interviewees in the three service types with a similar frequency. Clear guidelines were suggested in 10 countries, education programmes in 14 countries, positive relationships in 15 countries, and all other good practice components in all 16 countries.

## Discussion

### Main findings

In interviews with health care professionals in different types of services across Europe, eight problem areas were identified in the delivery of care to migrants. Seven components of good practice were suggested to overcome these problems or limit their potentially negative impact. Problem areas and good practice components are comprehensive, i.e. they covered all statements made in 240 interviews in 16 countries. Most problems and good practice components were raised in all countries and in more than 50% of the interviews, although the specific aspect and emphasis for each theme often varied among interviews. Problems and good practice components may therefore be seen as valid across Europe and different types of health services. The study findings provide a systematic and inclusive picture of the difficulties in providing health care to migrants and the possible solutions.

Interviewees were mindful of migrant specific difficulties and barriers, and believed that care to migrants was complicated by issues of language, culture, attitudes, entitlements, lack of awareness, and experience. The good practice components may be seen as more general, and several of them may apply not only to migrants, but to wider groups of patients. This reflects the overriding position of professionals that good practice in service delivery should be achieved by dispensing care on an individual basis, considering personal need, rather than focusing on group stereotypes and customary notions that migrants needs may differ greatly from indigenous patients. The findings provide support for treating migrant patients in mainstream health care services rather than segregating them out.

Good practice for migrants however may require additional and specific efforts. These include altering service delivery with modifications of routine practice, such as giving patients' with language needs more time, or seeking collaboration with social services that would be able to assist in legal and/or social issues. Problems and good practice components were largely consistent across the three very different types of services, i.e. primary care, emergency hospital departments, and community mental health services for patients with long term disorders.

### Strengths and Limitations

The study has a number of strengths: a) It is a large study using similar methods across 16 countries; b) the findings are based on a total of 240 interviews from countries with different histories of immigration so that saturation of findings can be assumed; c) we used similar selection criteria and the same interview schedule (apart from the origin of migrants in case vignettes) for all countries; d) we implemented a rigorous and consistent method for the analysis of the material which - to our knowledge - is unique for a qualitative study involving so many countries and languages, and the process was consistently quality managed; and e) the interview schedule was specific to health care delivery rather than the more often studied issue of access.

There are however also several limitations: a) the sampling frame for selecting areas was not strictly adhered to in all countries, and whilst most of the areas were in capitals this does not apply to all of them; b) interviewees were self-selected which may have introduced a bias, e.g. preferring professionals with a particular interest in migrant health and with specifically positive or critical views; c) statements may have been influenced by response tendencies, e.g. in line with the social desirability of answers, and reflect only personal statements; we have no independent information about what actually happens in the participating services and do not know the view of the migrant patients; and d) translations into English were required for the final analysis, information may have been lost in translation as a consequence.

### Comparisons with the literature

The problems areas in providing care to migrants identified in this study are consistent with those found in previous research. In a review of migrant health in the EU [13], language and literacy barriers, entitlement issues, cultural differences in expectations, and misunderstanding of Western medicine were suggested as impeding the access to and delivery of optimal health care. A negative impact of social migration stressors on both mental and physical health and migrant specific needs stemming from a lack of economic resources and limited social networks were noted in the literature on labour migrants and refugees [10,23,24].

Language barriers and the use of interpreters were issues mentioned by almost all respondents in this study and are commonly covered in the literature on migrant health care. Qualitative studies with refugees and asylum seekers in the United Kingdom highlighted the concerns patients had in trusting professional interpreters to maintain confidentiality [25]. Studies from the United States mirror the concern in using interpreters, even professional ones, as errors were documented in interpretations that entailed real clinical consequences, such as misdiagnosis and inadequate treatment [26].

Language barriers, different cultural norms, and different understandings of illness and treatment were identified as separate themes in this study, whilst in the literature they are often linked as obstacles to care experienced by both patients and practitioners alike [27,28]. Recommendations and interventions have tried to bridge the intercultural gap, with results from a randomised control trial in the Netherlands indicating improvement in mutual understanding after six months [29]. Overall, cultural differences appear to receive less attention than language barriers, and may be less considered in consultations with migrant patients [30]. Suggested ways forward include an increased awareness among practitioners of cultural differences and the use of advocates (not just interpreters) to increase mutual understanding without challenging more entrenched cultural beliefs [31-33].

A wider concern is the implication that cultural sensitivity might give rise to individual migrants being treated by ethnic group and by letting cultural expectations exceed individual preference. Discrimination and xenophobia in services were reported as a problem in providing care to migrants [34-36], which relates to the problem theme of negative attitudes in staff and patients in this study.

### Implications for improving practice

The three most frequent components of good practice, i.e. organisational flexibility with sufficient time and resources, good interpreting services, and working with families and social services, all related to changes at the organisational level and the availability of sufficient resources. Changes of organisational procedures, allocation of more time to patients with interpreting needs, and involving other services in dealing with social and legal affairs, would free practitioners to focus on delivering effective health care for all. This requires flexibility within the service and a willingness to collaborate with families and other services [37].

Other components of good practice advocated in this study require practitioner training and the provision of information to both health care staff and migrants. This applies to increasing cultural awareness amongst staff, providing educational programmes and information material for migrants, and circulating clear guidelines for staff on the care entitlements of different migrant groups. Previous research [5,9,10] suggests that knowledge of the health care system and awareness through experience may change the way migrants understand and utilise services, resulting in a more appropriate use of non-emergency services. Training and educational programmes may reduce the time it takes for this transition of service use to occur.

Attitude changes were also reported in this study as crucial to the delivery of good care. Where views of migrants, and of practitioners, were considered entrenched in stereotypes and unrealistic expectations, health care was seen as negatively affected. Positive relationships with staff and continuity of care were regarded as parts of good practice and helpful for combating negative attitudes towards care staff and migrant patients alike. Setting reasonable expectations for what services and practitioners can deliver may also reduce migrant patients' notable disappointment with services [15].

## Conclusions

The findings show and reflect a rich experience of health services across Europe in providing health care for migrant patients. There is a wide agreement on the relevant challenges and problems, which are not necessarily linked to the specific origin of the migrant. Health care professionals also have experiences and views about what constitutes good practice. The extent to which all good practice components are implemented varies, and there is certainly a chance for services to learn from each other and utilise experiences gained in other countries.

Implementing all good practice components as identified in this study requires sufficient resources, organisational flexibility, positive attitudes, training for staff, and the provision of information. The provision of sufficient resources, e.g. for more practitioner time and good interpreting services, is a challenge for commissioners and funding agencies, and is likely to be influenced by political priorities. Organisational flexibility does not always depend on the provision of more resources and may partly be achieved through appropriate policies and protocols. Training of staff also absorbs resources, and needs both the availability of effective training programmes and the interest of the staff to be trained. Information material should not be too difficult and costly to produce, although more evidence is required for how best to design and disseminate such material. The most challenging aspect to influence is probably staff attitudes, which may be linked to personal experiences as much as the wider societal context.

With respect to further research, the study shows that qualitative material can be collected in a consistent way across many countries and that an analysis of such material can yield useful findings. Future observational studies may be less comprehensive and address more narrowly defined aspects, capture patient views, assess actual behaviour in the services, and focus on service models that are regarded as very good practice. Experimental studies may test the feasibility and effectiveness of specific interventions to achieve one or more of the good practice components identified in this study.

## Competing interests

The authors declare that they have no competing interests.

## Authors' contributions

SP, SD, AG, TG, EI, UK, AK, ML, VL, RPR, AS, JJFS, MS, CS, KW, MW and MB all made substantial contributions to the design of the interview study, data collection, coding and initial stages of analysis, interpretation of the findings and critical revision of drafts. MB further coordinated the analysis across countries and centralised it in the UK. SS contributed to the analysis of findings, and drafting of the manuscript for publication.

## Pre-publication history

The pre-publication history for this paper can be accessed here:

http://www.biomedcentral.com/1471-2458/11/187/prepub
